# Sex Differences in Salivary Free Insulin-Like Growth Factor-1 Levels in Elderly Outpatients

**DOI:** 10.7759/cureus.17553

**Published:** 2021-08-30

**Authors:** Yoshinobu Yasuda

**Affiliations:** 1 Internal Medicine- Gastroenterology, Yasuda Clinic, Hamamatsu, JPN

**Keywords:** sex-based differences, salivary figf-1, salivary gh, serum igf-1, serum gh

## Abstract

Objective

Many studies have explored serum insulin-like growth factor (IGF)-1; however, only a few studies have investigated its presence in the saliva. Therefore, this study examined sex-based differences in salivary-free insulin-like growth factor-1 (fIGF-1), salivary growth hormone (GH), serum IGF-1 levels, and serum GH levels in older adults aged ≥60 years. The participants were further divided into <75 years and ≥75 years and examined the differences in the levels of the biomarkers mentioned above based on their sex.

Design

The participants were 80 regular outpatients (40 men and 40 women) with various diseases, including hypertension, diabetes, and hyperlipidemia. We randomly selected them based on the disease being treated. Men and women were divided into two groups according to age (aged <75 years or ≥75 years) for statistical analysis, including Student's t-test and Pearson's and Spearman's correlation coefficient tests.

Results

The analysis of sex differences in salivary fIGF-1 levels in patients aged <75 years showed significantly higher levels in women than in men. Correlation analyses of salivary fIGF-1 levels with salivary GH, serum IGF-1, and serum GH revealed a significant positive correlation of salivary fIGF-1 levels with serum IGF-1 and GH levels in men aged <75 years. In women aged ≥75 years, serum GH levels revealed a significant positive correlation with salivary GH levels and age.

Conclusions

The results suggested a higher possibility of the local synthesis of oral IGF-1 in women aged <75 years than in men aged <75 years.

## Introduction

The process of ageing differs according to sex. Sex-based differences in longevity have been studied; however, salivary biomarkers as indicators of the ageing process have not been studied. Kobayashi et al. used mouse submandibular glands to examine the age-related reduction in salivary insulin-like growth factor-1 (IGF-1) levels [1]. However, no studies have investigated the effects of ageing on human salivary IFG-1 with sex.

Compared with studies using blood samples, many researchers have pointed out that saliva samples have limitations such as stability issues and contamination by impurities [2,3]. However, Cabras et al. showed that salivary proteins are important markers of human physiology [4]. It has been reported that approximately 99% of blood IGF-1 binds to a specific binding protein and exists mainly as a trimer bound to IGF-binding protein 3 (IGFBP-3), which is almost saturated [5]. Conversely, approximately 1% of IGF-1 circulates in the blood as free IGF-1 [5] and is bioactive. On the other hand, Costigan et al. showed that almost all salivary IGF-1 is free IGF-1 [6].

Previous studies on salivary IGF-1 levels in childhood or middle age revealed that salivary IGF-1 levels increase in puberty and decrease after that [7]. However, there is no study on the age-related changes in salivary IGF-1 levels in older adults aged ≥60 years. In the current study, we examined the effects of sex-based differences in salivary fIGF-1 levels. We hypothesized that there are sex-based differences in human fIGF-1 levels regulated by the pituitary growth hormone (GH) and that the levels of fIGF-1 decrease with age.

## Materials and methods

Participants

This study enrolled outpatients with diseases treated at the author's clinic (Table 1). The survey period was from September to November 2019, and participants who were smokers or had saliva production of ≤1 mL were excluded. To perform statistical analysis on each test item, the male and female participants were divided into two groups according to age (aged <75 or ≥75 years). The participants were randomly selected and assigned to examination slots at 2:00 p.m. and 2:30 p.m. After a full explanation of the purpose of the test, all the participants provided written informed consent. When informed consent was obtained from the patients, they were apprised of the study approval by the Ethics Committee of Hamamatsu University School of Medicine (approval number: 19-010) and of their privacy protection according to the Declaration of Helsinki. In addition, after checking their medical history, we performed an intraoral assessment to examine the presence of any dental caries, prostheses, and defects.

**Table 1:** Patient characteristics

	Aged <75 years (n= 33)		Aged ≥75 years (n= 47)	
	Women (n= 15)	Men (n=18)	Women (n=25)	Men (n=22)
Hypertension (n)	9	7	10	12
Hyperlipidemia (n)	1	1	2	2
Diabetes mellitus (n)	0	1	0	1
Hypertension + hyperlipidemia (n)	1	4	7	3
Hypertension + diabetes mellitus (n)	2	2	1	3
Hyperlipidemia + diabetes mellitus (n)	0	0	1	0
Hypertension + hyperlipidemia + diabetes mellitus (n)	2	2	2	0
Other (n)	0	1	2	1
Mean age (years)	68.80	70.83	81.40	80.50

The saliva was collected using a stationary low-pressure suction device (SEASTER, Addex Co. Ltd., Bangkok, Thailand) with a power cable. We collected ≥1 mL of a saliva sample. The collection duration was set to 5 min whenever possible, and it was extended by 1 to 2 min only when the volume of the saliva collected was <1 mL. Using a Safeed aspiration catheter with a trap (Type P, TERUMO, Tokyo, Japan), the trap was put in a beaker containing ice water. Salivary secretion was promoted by letting the patients chew a 16-Fr flexible plastic tube. When the required amount of saliva was collected, the samples were transferred into plastic centrifuge tubes (15 mL; Model ECK-15ML; AS-1 Co., Osaka, Japan) (gamma radiation processed) and immediately underwent centrifugation at 8000 g for 5 min at 4°C. After centrifugation, 0.5 mL of saliva was dispensed into BM-ring lock tubes (gamma radiation process) and then stored in a special freezer at −80 °C. The human fIGF-1/IGF-1 ELISA Kit (Lot number P211212; R&D Systems, Minneapolis, MN, USA) and Human Growth Hormone Elisa Kit (Lot number P209747; RandD Systems, Minneapolis, MN, USA) were used for the measurements of salivary fIGF-1 and GH levels, respectively. All the samples were measured in duplicates, and the average value was adopted.

Statistical analysis

After confirming the normal distribution of the data, statistical analysis was performed using t-test and correlation analysis. Serum GH and salivary fIGF-1 levels showed skewed distributions. The results were analyzed following a natural logarithmic transformation of both data [8], and the level of significance was set at 5%. The statistical analysis was performed using four-step Excel Statistics (OMS Publishing, Saitama, Japan) [8].

## Results

**Figure 1:**
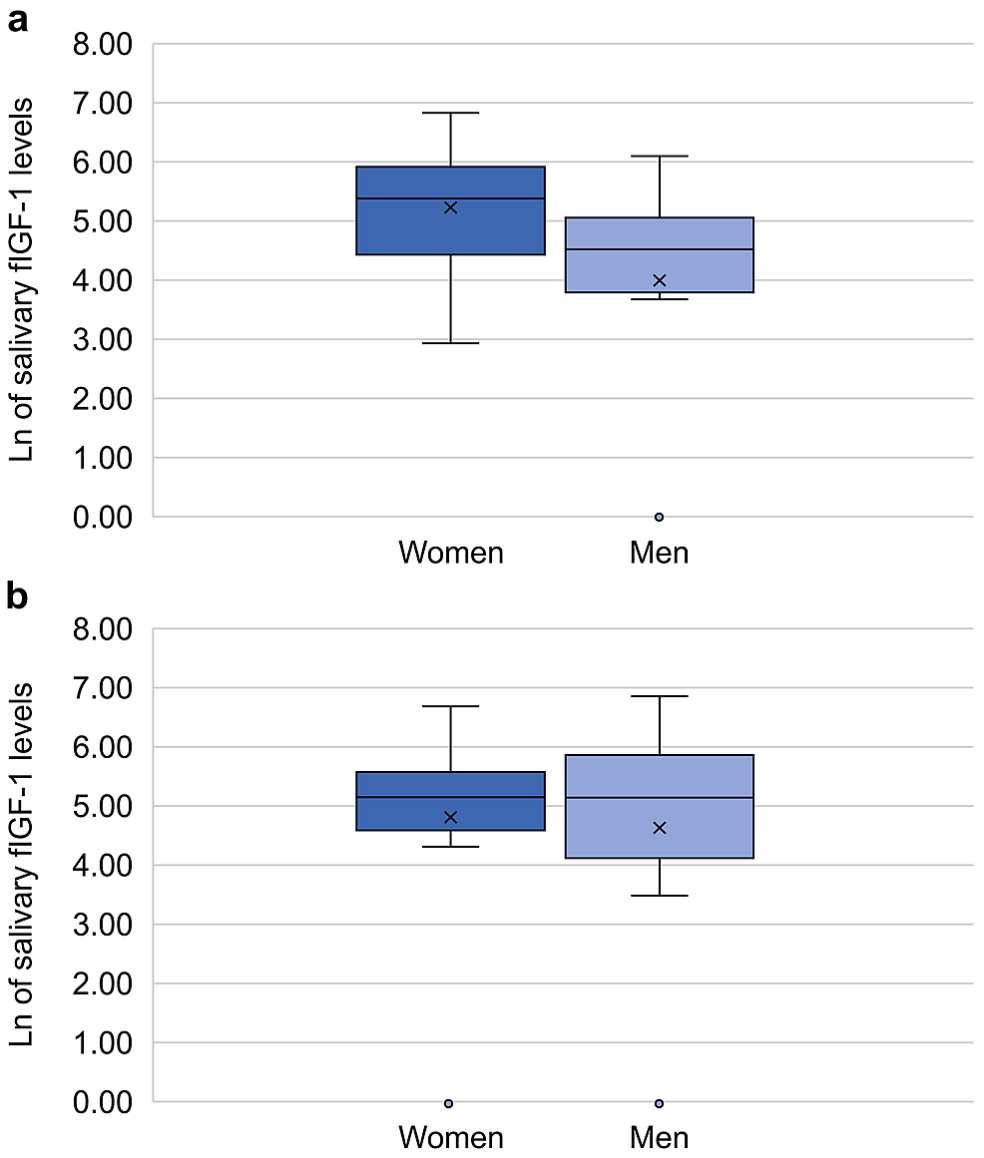
Ln of salivary fIGF-1 levels in women and men (a). Sex differences in salivary free insulin-like growth factor-1 (fIGF-1) levels in the participants aged <75 years. In women aged <75 years, the mean fIGF-1 level was 5.245 ± 1.062. In men aged <75 years, the mean fIGF-1 level was 4.009 ± 1.965. The mean fIGF-1 level was significantly higher in women than in men. Mann-Whitney U test = 191.5, p value=0.041 < 0.05. (b). Sex differences in salivary fIGF-1 levels in the participants aged ≥75 years. There were no significant sex differences in fIGF-1 levels in participants aged ≥75 years. Ln, natural logarithm

**Figure 2:**
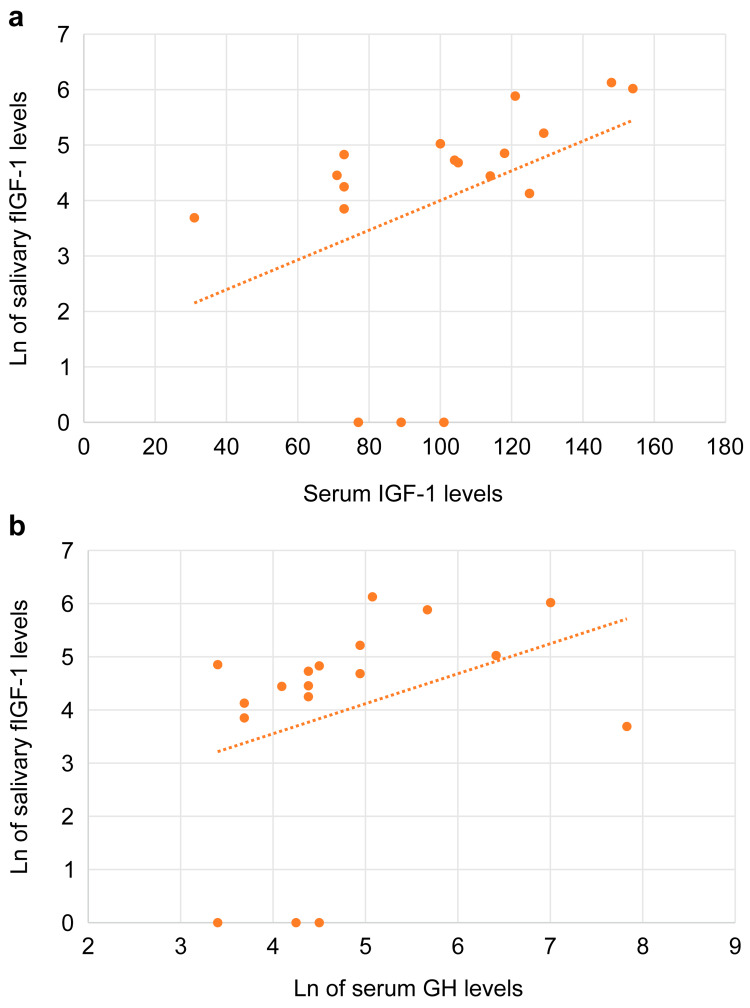
Correlation between Ln of salivary fIGH-1 levels and serum IGF-1 levels (a), Ln of serum GH levels (b) in men (a) Correlation between salivary fIGF-1 and serum IGF-1 levels in men aged <75 years. Tie-corrected Spearman’s rank correlation between salivary fIGF-1 and serum IGF-1 levels: rs = 0.623 (p < 0.05), where the value of rs needed to reject the null hypothesis was 0.472. (b) Correlation between salivary IGF-1 and serum GH levels in men aged <75 years. Tie-corrected Spearman's rank correlation between salivary IGF-1 and serum GH levels: rs = 0.501 (p < 0.05), where the value of rs needed to reject the null hypothesis was 0.472. Both subpanels a and b show a significant positive correlation. fIGF-1, free insulin-like growth factor-1; GH, growth hormone; Ln, natural logarithm; rs; Spearman’s correlation coefficient

Of the 80 participants enrolled in the study, 40 were male, and 40 were female. The analysis of sex differences in salivary fIGF-1 levels in the two groups (aged <75 years or ≥75 years) showed significantly higher salivary fIGF-1 levels in women aged <75 years (p < 0.05; Figure 1a, 1b) than in men in the same age group. By contrast, no sex differences were found for salivary GH levels. A correlation analysis exhibited a significant positive correlation of salivary fIGF-1 levels with serum IGF-1 and GH levels in men aged <75 years (Figures 2a, 2b). Salivary fIGF-1 levels showed a positive correlation with age in women aged <75 years and a negative correlation with age in women aged ≥75 years without any statistical significance.

 

**Figure 3:**
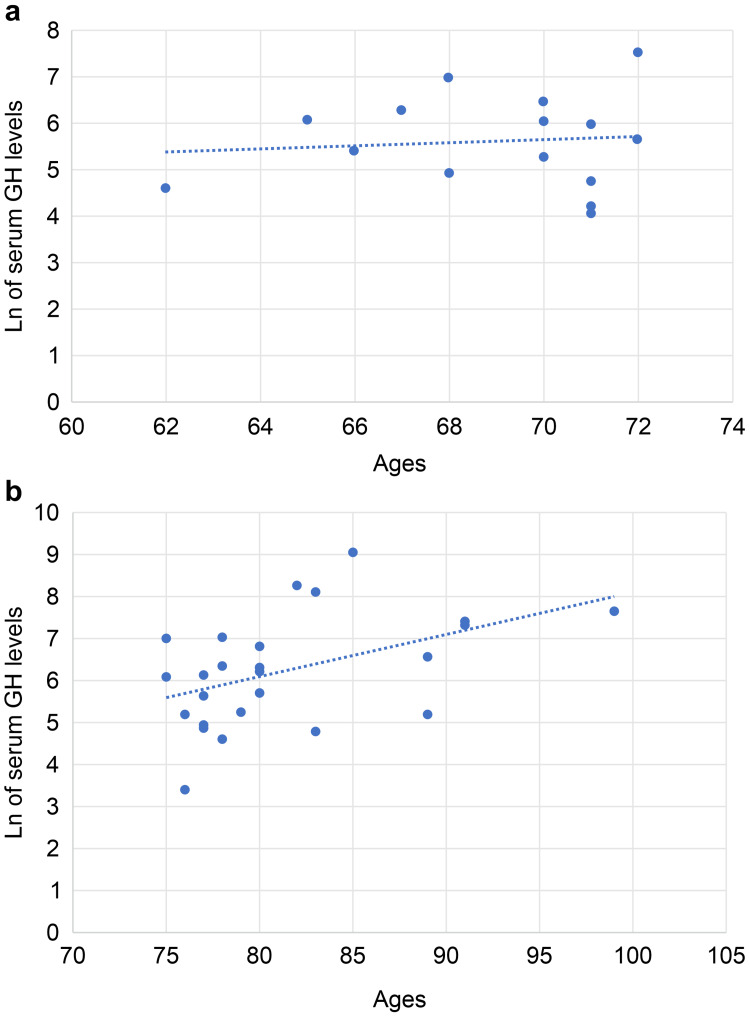
Correlation between serum GH levels and age in women (a) Correlation between serum growth hormone (GH) levels and age in women aged <75 years There was a positive but non-significant correlation between the serum GH concentration and age in patients aged <75 years (tie-corrected Spearman's rank correlation coefficient (rs) = 0.123, p < 0.05, where the rs value did not need to reject the null hypothesis was 0.485). (b) Correlation between serum GH levels and age in women aged ≧75 years. There was a positive significant positive correlation between serum GH concentration and age in patients aged ≧75 years (tie-corrected Spearman’s rank correlation coefficient rs=0.515, p<0.05, where the rs value needed to reject the null hypothesis was 0.398). Ln, natural logarithm

**Figure 4:**
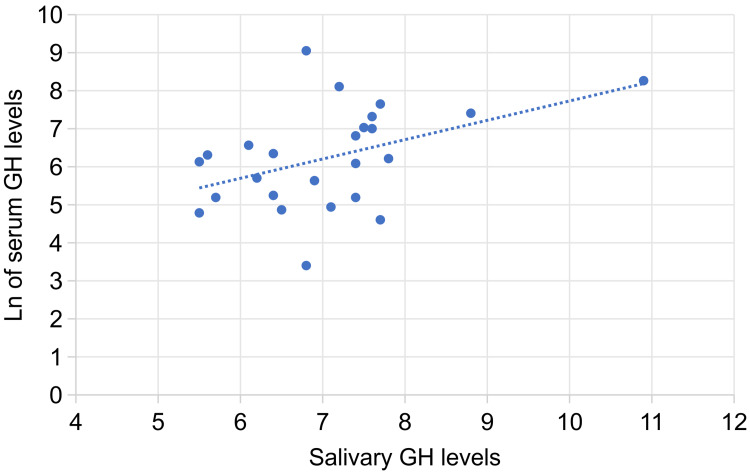
Correlation between salivary and serum GH levels in women aged ≥75 years Correlation between salivary and serum growth hormone levels in women aged ≥75 years Tie-corrected Spearman's rank correlation between salivary growth hormone (GH) and serum GH levels: rs = 0.413 (p < 0.05), where the rs value needed to reject the null hypothesis was 0.398, showing a significant positive correlation. GH: growth hormone; Ln: natural logarithm; rs: Spearman’s correlation coefficient.

Serum GH levels revealed a positive correlation with age in women aged <75 years without any statistical significance. However, in women aged ≥75 years, there was a significant positive correlation between serum GH levels and age (Figures 3a, 3b). In both groups (aged <75 or ≥75 years), there was a positive but non-significant correlation between salivary GH levels and age. There was a significant positive correlation between serum and salivary GH levels only in women aged ≥75 years (Figure 4).

 

## Discussion

In this study, the results suggest the progression of age-related oral frailty in women, as Tanaka et al. [9] reported. Correlational analyses between salivary fIGF-1 levels and other variables revealed a significant positive correlation between salivary fIGF-1 levels and serum GH and IGF-1 levels only in men aged <75 years. Salivary fIGF-1 in men aged <75 years is thought to be transferred primarily from the circulating blood to the saliva [10]. However, there was no significant correlation between the two variables in women of the same age group. It can speculate that sex differences are due to the local synthesis of fIGF-1 from the following factors, submandibular glands [11] and tooth-forming cells [12], such as periodontal ligament cells. The mechanism of local synthesis may be the most significant contributor toward salivary fIGF-1 secretion in women aged <75 years.

It has been reported that the degree of correlation between serum GH levels and age generally becomes weaker after 60 years of age [13]. However, in the present study, serum GH levels showed a significant positive correlation with age in women aged ≥75 years. The results are consistent with the study conducted on anorexia nervosa by Gianotti et al. [14], who concluded that serum IGF-1 levels decrease. As a form of negative feedback, serum GH levels increase in anorexia nervosa to cope with chronic malnutrition. This suggests the rapid age-related progression of malnutrition in women.

Furthermore, this study revealed a significant positive correlation between serum and salivary GH levels in women aged ≥75 years (Figure 4). The results suggest the presence of salivary GH not originating from the circulating blood but locally synthesized [15] and the paracrine effects of the GH/IGF-1 axis measured in saliva from the oral cavity.

## Conclusions

The sex differences may be due to the paracrine effects of the GH/IGF-1 axis measured in saliva from the oral cavity. Future studies should elucidate the mechanisms involved in the sex differences in salivary fIGF-1 levels due to the paracrine effects of the GH/IGF-1 axis measured in saliva from the oral cavity and ageing.

## References

[1] Kobayashi S, Kamino Y, Hiratsuka K, Kiyama-Kishikawa M, Abiko Y (2004). Age-related changes in IGF-1 expression in submandibular glands of senescence-accelerated mice. J Oral Sci.

[2] Tenovuo JO (1998). Human Saliva: Clinical Chemistry & Microbiology, volumes 1 and 2.

[3] Schipper RG, Silletti E, Vingerhoeds MH (2007). Saliva as research material: biochemical, physicochemical and practical aspects. Arch Oral Biol.

[4] Cabras T, Pisano E, Boi R (2009). Age-dependent modifications of the human salivary secretory protein complex. J Proteome Res.

[5] Monzavi R, Cohen P (2002). IGFs and IGFBPs: role in health and disease. Best Pract & Res Clin Endocrinol and Metab.

[6] Costigan DC, Guyda HJ, Posner BI (1988). Free insulin-like growth factor 1 (IGF-1) and IGF2 in human saliva. J Clin Endocrinol Metab.

[7] Ryan J, Mantle T, Costigan DC (1991). A normal population study of human salivary insulin-like growth factor 1 (IGF-1) concentrations from birth through puberty. J Clin Endocrinol Metab.

[8] Yanai H (2018). Four-Step Excel Statistics.

[9] Tanaka T, Takahashi K, Hirano H (2018). Oral frailty as a risk factor for physical frailty and mortality in community-dwelling elderly. J Gerontol.

[10] Fallo F, Maffei P, Dalla Pozza A (2009). Cardiovascular autonomic function in Cushing's syndrome. J Endocrinol Invest.

[11] Ryan J, Mantle T, McQuaid S, Costigan DC (1992). Salivary insulin-like growth factor-I originates from local synthesis. J Endocrinol.

[12] Werner H, Katz J (2004). The emerging role of the insulin-like growth factors in oral biology. J Dent Res.

[13] Perice L, Barzilai N, Verghese J, Weiss EF, Holtzer R, Cohen P, Milman S (2016). Lower circulating insulin-like growth factor-I is associated with better cognition in females with exceptional longevity without compromise to muscle mass and function. Aging (Albany NY).

[14] Gianotti L, Lanfranco F, Ramunni J, Destefanis S, Ghigo E, Arvat E (2002). GH/IGF-I axis in anorexia nervosa. Eat Weight Disord.

[15] Tresguerres JA, Ariznavarreta C, Granados B, Costoya JA, Pérez-Romero A, Salamé F, Hermanussen M (1999). Salivary gland is capable of GH synthesis under GHRH stimulation. J Endocrinol.

